# West Nile Morbidity and Mortality in a Mid-Atlantic Health Care System, 2013–2024

**DOI:** 10.4269/ajtmh.26-0008

**Published:** 2026-04-09

**Authors:** Seth D. Judson, Paul Auwaerter, David Dowdy

**Affiliations:** ^1^ Division of Infectious Diseases, Department of Medicine, David Geffen School of Medicine at UCLA, Los Angeles, California, USA;; ^2^ Division of Infectious Diseases, Department of Medicine, Johns Hopkins University School of Medicine, Baltimore, Maryland, USA;; ^3^ Sherilyn and Ken Fisher Center for Environmental Infectious Diseases, Johns Hopkins University School of Medicine, Baltimore, Maryland, USA;; ^4^ Department of Epidemiology, Johns Hopkins Bloomberg School of Public Health, Baltimore, Maryland, USA

## Abstract

West Nile virus (WNV) is transmitted by mosquitoes to humans in many regions and can cause neuroinvasive disease and death. To better understand the local burden of disease caused by WNV and risk factors for disease severity, we analyzed cases from across the Johns Hopkins Health System Corporation. We identified 86 patients who met confirmed/probable case definitions for WNV infection from 2013 to 2024. Seventy-one patients (83%) had West Nile neuroinvasive disease (WNND), and nine (10%) died. Encephalitis (*n* = 47) was the most common complication and was associated with increased age, alcohol use disorder, immunocompromised status, and a higher Charlson Comorbidity Index. Of the 21 patients in the cohort who were immunocompromised, 20 (95%) developed WNND, and 4 (19%) died. The overall median hospital length of stay was 8 days (interquartile range 5–15). People diagnosed with WNV in this health care system experienced significant morbidity and mortality from WNND, notably when immunocompromised.

## INTRODUCTION

West Nile virus (WNV) causes the most significant morbidity and mortality of any vector-borne virus in North America.^1^ There are currently no treatments or vaccines for WNV in humans; therefore, preventing infection is the primary method for mitigating disease. Understanding the local disease burden and risk factors for severe WNV disease can help clinicians and public health practitioners develop an appropriate response.

Approximately 25% of patients infected with WNV develop a febrile syndrome called West Nile fever (WNF), whereas <1% develop neuroinvasive disease (WNND).^2^^,^^3^ The main manifestations of WNND include meningitis (WNM), encephalitis (WNE), and acute flaccid paralysis (AFP).^4^ The spectrum of WNND may overlap and can result in lifelong neurological sequelae and death, with a case fatality ratio ∼10%.^1^

Although many reports of the burden and risk factors for WNND in North America exist, most of these studies primarily involve patients from the western and midwestern United States.^5^^–^^7^ Considering the heterogeneity of patient populations, the increasing prevalence of immunosuppression, and novel data available in electronic medical records (EMRs), contemporary analyses of WNND are needed.^8^ A recent meta-analysis found no studies of WNND from Maryland, Virginia, or Washington, DC.^7^ The Johns Hopkins Health System Corporation (JHHS) cares for many patients from these Mid-Atlantic jurisdictions. Therefore, we aimed to characterize the burden of, and risk factors for, severe WNND and WNV mortality from 2013 to 2024 within the JHHS.

## MATERIALS AND METHODS

### Cohort selection.

The JHHS includes more than 40 outpatient facilities and 6 hospitals in Maryland; Washington, DC; and Florida (which has only pediatric facilities and was excluded from this analysis). Data were extracted from the Epic Clarity database that includes both inpatient and outpatient EMRs using search criteria for patients with the *International Statistical Classification of Diseases, Tenth Revision* (*ICD-10)* codes for WNV infection (A92.30, A92.31, A92.32, A92.39) or positive WNV diagnostic results (WNV IgG, IgM, or polymerase chain reaction [PCR]). Data were accessed on April 24, 2025. The final cohort included cases that occurred between January 1, 2013 (the year that Epic was launched at JHHS) and December 31, 2024. All records identified with positive *ICD-10* codes and/or laboratory results for WNV underwent chart review for inclusion using the CDC’s WNV neuroinvasive and non-neuroinvasive probable/confirmed case definitions to identify cases of WNND and WNF.^9^ Inclusion criteria included adults (≥18 years of age) diagnosed with laboratory-established WNV infection (positive serum and/or cerebrospinal fluid (CSF) WNV IgM or PCR) in the setting of compatible clinical history between 2013 and 2024. Clinical signs/symptoms, laboratory results, and imaging were used to classify patients as having WNF and WNND, defined as WNE, WNM, and/or AFP (Supplemental Table 1; Supplemental Figure 1).

### Outcomes and covariates.

Our primary outcome for morbidity was WNND. Given that WNE is the most prevalent severe form of WNND, we assessed risk factors for WNE in a secondary analysis.

Our co-primary outcomes for mortality were all-cause mortality and mortality directly attributable to WNV. Our secondary outcomes included hospitalization, intensive care unit (ICU) admission, and hospital length of stay (LOS).

We assessed potential risk factors with a priori evidence of increased risk for WNND and mortality: age, sex, cancer, acute myocardial infarction, congestive heart failure, diabetes, chronic kidney disease (CKD), liver disease, cerebrovascular disease, chronic pulmonary disease, multiple sclerosis, dementia, rheumatic disease, peripheral vascular disease, hypertension, alcohol use disorder, and immunosuppressant use.^5^^,^^6^^,^^8^^,^^10^ We also calculated the Charlson Comorbidity Index (CCI) for each patient as a composite risk factor (excluding age from the calculation). The CCI was calculated using the *ICD-10*–based scoring system (Supplemental Table 2).^11^ Both individual risk factors and CCI components were assessed at the date of the encounter in which WNV was first reported. We also evaluated immunocompromised status as a risk factor, defined as one or more of the following: history of solid organ transplant, active malignancy, HIV with CD4 count <200, immunosuppressant use (calcineurin inhibitors, high-dose prednisone, mycophenolate, and/or biologics), or receiving chemotherapy.

## STATISTICAL ANALYSES

We calculated descriptive statistics for our primary and secondary outcomes. In evaluating risk factors for WNE, we first used logistic regression to assess bivariate associations with each outcome. Covariates with a *P*-value <0.2 and present in more than five patients total were entered into a multivariable logistic regression model, and stepwise backward selection was then used to identify the model with the optimal Akaike information criterion (AIC) value using the MASS package in R (R Foundation for Statistical Computing) (Supplemental Figure 1). Given the potential for collinearity between covariates and the CCI, a separate multivariable model containing age, sex, and CCI was created. A two-sided *P*-value <0.05 was used for statistical significance for the final covariates. All statistical analyses were conducted in R v. 4.4.2.

## RESULTS

Among 367 adult patients with positive *ICD-10* codes and/or laboratory results for WNV, 86 patients met inclusion criteria as confirmed/probable cases. Most patients (78/86, 91%) were diagnosed with WNV during the period August through October.

### WNND.

Seventy-one of 86 patients (83%) had WNND, and 15 (17%) had WNF (Table 1). Of those with WNND, 47 (66%) had encephalitis/meningoencephalitis (i.e., WNE), 18 (25%) had meningitis without encephalitis, 4 (6%) had AFP (2 with AFP/WNE), and 2 (3%) had transverse myelitis. Most patients with WNND were male (47/71, 66%), were ≥50 years old (57/71, 80%), and had at least one CCI comorbidity (47/71, 66%).

**Table 1:** Characteristics of patients with WNV in a Mid-Atlantic health care system, 2013–2024

Covariate	Patients, No. (%)			
	WNND: WNE (*n* = 47)	WNND: Non-WNE (*n* = 24)	WNF (*n* = 15)	Total (*N* = 86)
Age at diagnosis, median (IQR), years	67 (59–76)	51 (43–65)	58 (45–63)	62 (51–71)
Sex				
Female	10 (21)	14 (58)	5 (33)	29 (34)
Male	37 (79)	10 (42)	10 (42)	57 (66)
Ethnicity				
Not Hispanic or Latino	45 (96)	22 (92)	13 (87)	80 (93)
Hispanic or Latino	1 (2)	1 (4)	2 (13)	4 (5)
Unknown ethnicity	1 (2)	1 (4)	0 (0)	2 (2)
Race				
White	40 (85)	17 (71)	11 (73)	68 (79)
Black or African American	5 (11)	2 (8)	1 (7)	8 (9)
Asian	1 (2)	1 (4)	0 (0)	2 (2)
Other/unknown race	1 (2)	4 (17)	3 (20)	8 (9)
Cancer	11 (23)	1 (4)	0 (0)	12 (14)
Acute myocardial infarction	11 (23)	0 (0)	1 (7)	12 (14)
Congestive heart failure	5 (11)	0 (0)	0 (0)	5 (6)
Diabetes Mellitus (Overall)	5 (11)	3 (13)	1 (7)	9 (11)
Diabetes without complications	2 (4)	2 (8)	1 (7)	5 (6)
Diabetes with complications	3 (6)	1 (4)	0 (0)	4 (5)
HIV (overall)	3 (6)	1 (4)	0 (0)	4 (5)
AIDS	2 (4)	1 (4)	0 (0)	3 (4)
Chronic kidney disease (overall)	8 (17)	0 (0)	1 (7)	9 (11)
Mild renal disease	2 (4)	0 (0)	1 (7)	3 (3)
Severe renal disease	6 (13)	0 (0)	0 (0)	6 (7)
Liver disease (overall)	6 (13)	2 (8)	0 (0)	8 (9)
Mild liver disease	5 (11)	2 (8)	0 (0)	7 (8)
Moderate/Severe liver disease	1 (2)	0 (0)	0 (0)	1 (1)
Cerebrovascular diseases	5 (11)	0 (0)	0 (0)	5 (6)
Chronic pulmonary disease	7 (15)	3 (13)	0 (0)	10 (11)
Multiple Sclerosis	1 (2)	0 (0)	0 (0)	1 (1)
Dementia	4 (9)	0 (0)	0 (0)	4 (5)
Rheumatic disease	2 (4)	1 (4)	1 (1)	4 (5)
Peripheral vascular disease	6 (13)	0 (0)	1 (7)	7 (8)
Alcohol use disorder	7 (15)	1 (4)	0 (0)	8 (9)
Hypertension	22 (47)	6 (25)	5 (33)	33 (38)
Transplanted organ	7 (15)	0 (0)	0 (0)	7 (8)
Immunocompromised	16 (34)	4 (17)	1 (7)	21 (24)
Immunosuppressants (overall)	13 (28)	3 (13)	1 (7)	17 (20)
Calcineurin inhibitor	6 (13)	0 (0)	0 (0)	6 (7)
Mycophenolate	5 (11)	1 (4)	0 (0)	6 (7)
Prednisone	4 (9)	0 (0)	0 (0)	4 (5)
Ocrelizumab or Rituximab	3 (6)	0 (0)	0 (0)	3 (3)
Charlson comorbidity index				
0	10 (21)	14 (58)	10 (67)	34 (40)
1–2 (mild)	17 (36)	7 (29)	5 (33)	29 (34)
3–4 (moderate)	10 (21)	2 (8)	0 (0)	12 (14)
>5 (severe)	10 (21)	1 (4)	0 (0)	11 (13)

IQR = interquartile range; WNE = West Nile encephalitis; WNF = West Nile fever; WNND = West Nile neuroinvasive disease; WNV = West Nile virus.

Of 21 immunocompromised patients, 20 (95%) developed WNND, including 16/17 (94%) on immunosuppressants (including calcineurin inhibitors, mycophenolate, prednisone, biologics [adalimumab, rituximab, ocrelizumab], and antineoplastics [venetoclax and acalabrutinib]), and 7/7 (100%) with history of solid organ transplant.

Potential risk factors for WNE (*P* <0.2) in the univariable analysis included age, male sex, cancer, acute myocardial infarction, CKD, peripheral vascular disease, hypertension, alcohol use disorder, immunosuppressant use, immunocompromised status, and CCI. The most parsimonious model (lowest AIC) included age (adjusted odds ratio [aOR] = 2.46 per 10 years; 95% CI = 1.64–4.05), CKD (aOR = 6.76; 95% CI = 0.76–175), alcohol use disorder (aOR = 11.8; 95% CI = 1.59–248), and immunocompromised status (aOR = 6.71; 95% CI = 1.55–38.2) (Supplemental Table 3). In the multivariable model including CCI, indices of 3–4 (aOR = 7.84; 95% CI = 1.28–75.1) and >5 (aOR = 19.9; 95% CI = 2.78–421) relative to an index of 0 were significantly associated with WNE (Supplemental Table 4). In both multivariable models, variance inflation factors were all below 2, indicating no significant collinearity, and the Hosmer-Lemeshow test demonstrated adequate fit (*P*-values 0.61 and 0.29).

### Hospitalization.

Of the 86 patients with WNV in the cohort, 74 (86%) were hospitalized, including 68/71 (96%) of patients with WNND and 6/15 (40%) with WNF. Of the 74 admitted patients, 26 (35%) required ICU admission (all with WNND), and 21 (28%) were immunocompromised (all immunocompromised patients were hospitalized). The overall median hospital LOS was 8 days (interquartile range 5–15).

### Mortality.

Nine of 86 patients (10%) with WNV infection from 2013 to 2024 died; all of these patients had WNE, and two also had AFP. Among these patients, 5 (55%) died within 30 days of admission, with WNV identified as the cause of death. All of those who died were >50 years old and had at least one CCI comorbidity; four (44%) were immunocompromised (Table 2).

**Table 2:** Characteristics of patients with WNV and mortality, 2013–2024

Covariate	Patients, *n* (%)		
	WNV and No Death (*n* = 77)	WNV and Death (*n* = 9)	Total (*N* = 86)
Age at diagnosis, median (IQR), years	61 (50–71)	68 (55–74)	62 (51–71)
Sex			
Female	28 (36)	1 (11)	29 (34)
Male	49 (64)	8 (89)	57 (66)
Ethnicity			
Not Hispanic or Latino	71 (92)	9 (100)	80 (93)
Hispanic or Latino	4 (5)	0 (0)	4 (5)
Unknown ethnicity	2 (3)	0 (0)	2 (2)
Race			
White	61 (80)	7 (78)	68 (79)
Black or African American	7 (9)	1 (11)	8 (9)
Asian	2 (3)	0 (0)	2 (2)
Other/unknown race	7 (9)	1 (11)	8 (9)
Cancer	12 (15)	0 (0)	12 (14)
Acute myocardial infarction	7 (9)	5 (55)	12 (14)
Congestive heart failure	3 (4)	2 (22)	5 (6)
Diabetes Mellitus (Overall)	9 (11)	0 (0)	9 (11)
Diabetes without complications	5 (7)	0 (0)	5 (6)
Diabetes with complications	4 (5)	0 (0)	4 (5)
HIV (overall)	4 (5)	0 (0)	4 (5)
AIDS	3 (4)	0 (0)	3 (4)
Chronic kidney disease (overall)	4 (5)	5 (56)	9 (11)
Mild renal disease	2 (3)	1 (11)	3 (3)
Severe renal disease	2 (3)	4 (44)	6 (7)
Liver disease (overall)	5 (7)	3 (33)	8 (9)
Mild liver disease	5 (7)	2 (22)	7 (8)
Moderate/Severe liver disease	0 (0)	1 (11)	1 (1)
Cerebrovascular diseases	2 (3)	3 (33)	5 (6)
Chronic pulmonary disease	8 (10)	2 (22)	10 (11)
Multiple Sclerosis	1 (1)	0 (0)	1 (1)
Dementia	4 (5)	0 (0)	4 (5)
Rheumatic disease	4 (5)	1 (4)	4 (5)
Peripheral vascular disease	5 (7)	2 (22)	7 (8)
Alcohol use disorder	6 (8)	2 (22)	8 (9)
Hypertension	28 (36)	5 (57)	33 (38)
Transplanted organ	3 (4)	4 (44)	7 (8)
Immunocompromised	17 (22)	4 (44)	21 (24)
Immunosuppressants (overall)	13 (17)	4 (44)	17 (20)
Calcineurin inhibitor	3 (4)	3 (33)	6 (7)
Mycophenolate	2 (3)	4 (4)	6 (7)
Prednisone	1 (1)	3 (33)	4 (5)
Ocrelizumab or Rituximab	3 (4)	0 (0)	3 (4)
Charlson comorbidity index			
0	34 (44)	0 (0)	34 (40)
1–2 (mild)	27 (35)	2 (22)	29 (34)
3–4 (moderate)	8 (10)	4 (44)	12 (14)
>5 (severe)	8 (10)	3 (33)	11 (13)

IQR = interquartile range; WNV = West Nile virus.

## DISCUSSION

We identified significant morbidity and mortality from WNV in a Mid-Atlantic health care system from 2013 to 2024. Most diagnosed patients had WNND, with WNE being the most common neurological manifestation, aligning with prior studies.^3^ The high proportions of hospitalization (86%), ICU admission (35%), and prolonged LOS (median 8 days) further illustrate the impact of WNV on the health care system.^12^ Of nine patients who died, WNV was identified as the cause of death in five—all of whom died within 30 days of diagnosis.

Our study reinforces the known association between increased age and WNND; the odds of WNE doubled with every decade of age in our cohort. We also found a high prevalence of WNND among immunocompromised individuals who were diagnosed with WNV. Immunocompromised status, including the use of various immunosuppressants, corroborates recent findings that immunosuppression is associated with WNND and its severity.^13^ Alcohol use disorder was also identified as a significant risk factor for WNE, consistent with prior studies.^6^^,^^14^

A limitation of this analysis is the inclusion of probable WNV cases that were diagnosed serologically without specific neutralizing antibody confirmatory testing and could represent cross-reactivity with other flaviviruses. Using CDC case definitions for acute (probable/confirmed) WNV infection also limited our sample size and statistical power. In this setting, we found that the CCI may be a useful tool for evaluating risk of WNE, because it can be readily calculated from clinical data when individual comorbidities are less prevalent. This cohort also had a higher proportion of WNND in comparison with the 2023 national average (83% versus 68%),^15^ indicating potential under-ascertainment of WNF and/or higher acuity of disease in this referral-level hospital system.

## CONCLUSION

In summary, our findings of substantial morbidity and mortality for WNV in a Mid-Atlantic health care system over the past decade highlight the need for increased surveillance, targeted prevention, and countermeasure development (including a WNV vaccine). These measures could be especially impactful for immunocompromised patients and older patients with multiple comorbidities.

## Supplemental Materials

10.4269/ajtmh.26-0008 Supplemental Materials
