# Supplementary material for: West Nile Morbidity and Mortality in a Mid-Atlantic Health Care System, 2013–2024
Source: Am J Trop Med Hyg. 2026 Apr 9;114(6):1212–5. doi: 10.4269/ajtmh.26-0008 (PMC13235596; doi:10.4269/ajtmh.26-0008)
Supplement: Supplemental Materials [file tpmd260008.SD1.pdf]

Supplementary Appendix

S Table 1. Diagnostic Criteria for West Nile Neuroinvasive Disease Outcomes

Diagnosis	Criteria
WNF	Positive serum IgM or PCR<br>AND<br>Any of the following clinical signs/symptoms:<br>Fever, myalgias, fatigue, rash, nausea/vomiting/diarrhea, headache<br>Cannot have: neurological signs/symptoms, or positive CSF IgM or PCR
WNM	Positive serum/CSF IgM or PCR<br>AND<br>Any of the following clinical signs/symptoms: meningeal inflammation (nuchal rigidity, neck pain, photophobia, headache), CSF pleocytosis, and/or consistent MRI imaging
WNE	Positive serum/CSF IgM or PCR<br>AND<br>Any of the following clinical signs/symptoms: Altered mental status, seizures, abnormal EEG, focal neurological deficits, and/or consistent MRI imaging
AFP	Positive serum/CSF IgM or PCR<br>AND<br>Any of the following clinical signs/symptoms: Acute onset of flaccid limb weakness or paralysis, consistent MRI imaging/EMG and/or examination and diagnosis of AFP by neurologist

Diagnostic testing in this cohort was primarily done via serological testing in the context that the Johns Hopkins Healthcare System removed the WNV CSF PCR from the test menu in 2018 favoring serological testing given sensitivity and cost-effectiveness, as referenced below.

Karaba AH, Blair PW, Martin K, Saheed MO, Carroll KC, Borowitz MJ., 2019. The Effects of a Systemwide Diagnostic Stewardship Change on West Nile Virus Disease Ordering Practices. Open Forum Infectious Diseases 6: ofz488

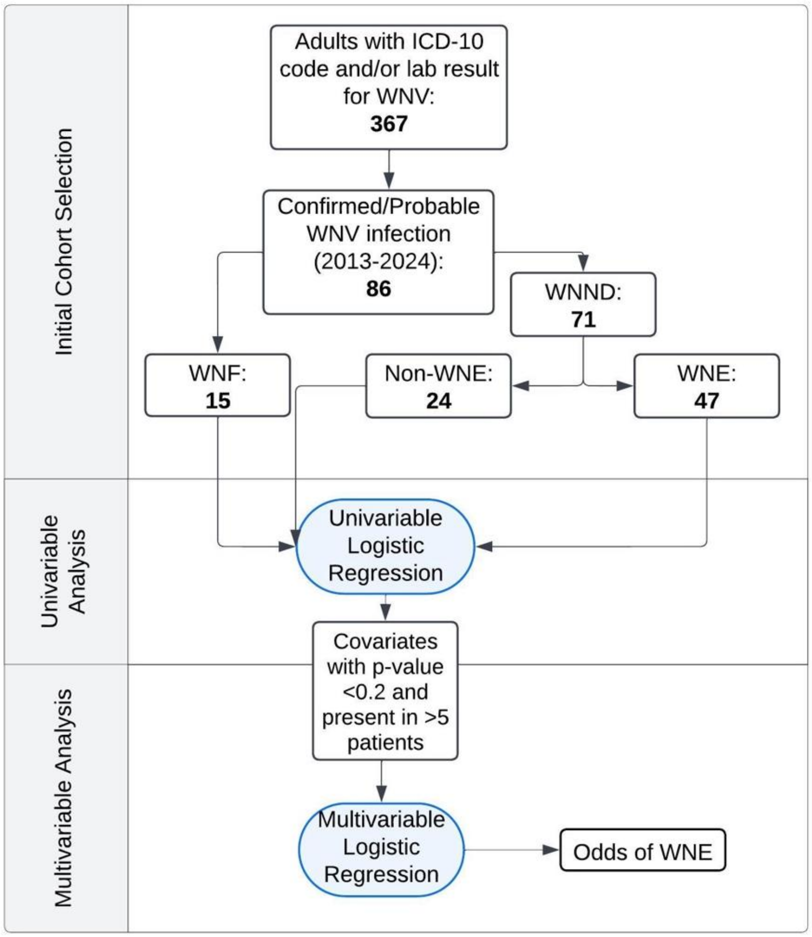

S Figure 1. Flow diagram of cohort selection and analysis

The diagram shows the cohort selection process and risk factor modeling analysis. The initial cohort included all adult patients within the JHHS Epic Clarity database with ICD-10 codes for WNV infection and/or positive WNV diagnostic results. Records underwent chart review for inclusion using the CDC's West Nile virus neuroinvasive and non-neuroinvasive probable/confirmed case definitions. The final cohort included confirmed/probable cases that occurred between January 1, 2013 and December 31, 2024. To assess risk for West Nile encephalitis (WNE), we used logistic regression to assess bivariate associations. Covariates with a p-value <0.2 and present in >5 patients total were entered into a multivariable logistic regression model, and stepwise backward selection was then used to identify the model with the optimal AIC (Akaike Information Criterion).

Supplementary Appendix

The Charlson Comorbidity Index was calculated using the scoring system and diagnoses described in the supplement of Glasheen WP, Cordier T, Gumpina R, Haugh G, Davis J, Renda A., 2019. Charlson Comorbidity Index: ICD-9 Update and ICD-10 Translation. *Am Health Drug Benefits 12*: 188–197

The table below shows the points allocation for each condition. If a patient had multiple diagnoses in the same category (i.e. Renal disease, severe vs. Renal disease, mild to moderate) the more severe diagnosis was used.

S Table 2. Charlson Comorbidity Index

Diagnosis	Points
Myocardial infarction	1
Congestive heart failure	1
Peripheral vascular disease	1
Cerebrovascular disease	1
Dementia	1
Chronic pulmonary disease	1
Rheumatic disease	1
Peptic ulcer disease	1
Liver disease, mild	1
Diabetes without chronic complications	1
Renal disease, mild to moderate	1
Diabetes with chronic complications	2
Hemiplegia or paraplegia	2
Any malignancy	2
Liver disease, moderate to severe	3
Renal disease, severe	3
HIV infection, no AIDS	3
Metastatic solid tumor	6
AIDS	6

S Table 3. Univariable and Multivariable Associations with WNE

Covariate	Unadjusted OR (95% CI)	Adjusted OR (95% CI)*	P-value†
Age per decade	1.93 (1.40 to 2.83)	2.46 (1.64 to 4.05)	<0.0001
Male sex	3.51 (1.40 to 9.28)	-	-
Cancer	11.6 (2.10 to 218)	-	-
Acute myocardial infarction	11.6 (2.10 to 218)	-	-
Chronic kidney disease	7.79 (1.34 to 148)	6.76 (0.756 to 175)	0.14
Peripheral vascular disease	5.56 (0.89 to 108)	-	-
Hypertension	2.24 (0.92 to 5.67)	-	-
Alcohol use disorder	6.65 (1.11 to 127)	11.84 (1.59 to 248)	0.035
Immunosuppressants	3.35 (1.06 to 12.8)	-	-
Immunocompromised	3.51 (1.21 to 11.8)	6.71 (1.55 to 38.2)	0.018

*OR= Odds Ratio; CI= Confidence Interval; Adjusted results based on multivariable logistic regression
†P-value for adjusted results

AIC of the initial model including all of the above covariates: 97.5

AIC of the final model using backwards selection (including age, CKD, alcohol use disorder, and immunocompromised): 90.8

Goodness-of-fit for final models was assessed using the Hosmer-Lemeshow test with five bins, and multicollinearity was assessed using Variance Inflation Factors (VIFs).

S Table 4. Univariable and Multivariable Associations of CCI with WNE

Covariate	Unadjusted OR (95% CI)	Adjusted OR (95% CI)*	P-value†
Age per decade	1.93 (1.40 to 2.83)	1.97 (1.35 to 3.16)	<0.0001
Male sex	3.51 (1.40 to 9.28)	2.07 (0.615 to 7.13)	0.24
CCI††			
0	Ref	Ref	Ref
1-2	3.40 (1.22 to 9.98)	2.06 (0.608 to 7.06)	0.24
3-4	12.0 (2.60 to 88.0)	7.84 (1.28 to 75.1)	0.04
>5	24.0 (3.86 to 471)	19.9 (2.78 to 421)	0.01

*OR= Odds Ratio; CI= Confidence Interval; Adjusted results based on multivariable logistic regression
†P-value for adjusted results
††CCI = Charlson Comorbidity Index
